# A simple cut and stretch assay to detect antimicrobial resistance genes on bacterial plasmids by single-molecule fluorescence microscopy

**DOI:** 10.1038/s41598-022-13315-w

**Published:** 2022-06-03

**Authors:** Gaurav Goyal, Elina Ekedahl, My Nyblom, Jens Krog, Erik Fröbrant, Magnus Brander, Tsegaye Sewunet, Teerawit Tangkoskul, Christian G. Giske, Linus Sandegren, Visanu Thamlikitkul, Tobias Ambjörnsson, Fredrik Westerlund

**Affiliations:** 1 Department of Biology and Biological Engineering, Chalmers University of Technology, Gothenburg, Sweden; 2 Department of Astronomy and Theoretical Physics, Lund University, Lund, Sweden; 3 Department of Laboratory Medicine, Karolinska Institute, Stockholm, Sweden; 4 Faculty of Medicine Siriraj Hospital, Mahidol University, Bangkok, Thailand; 5 Department of Clinical Microbiology, Karolinska University Hospital, Stockholm, Sweden; 6 Department of Medical Biochemistry and Microbiology, Uppsala University, Uppsala, Sweden

**Keywords:** Antimicrobial resistance, Molecular medicine

## Abstract

Antimicrobial resistance (AMR) is a fast-growing threat to global health. The genes conferring AMR to bacteria are often located on plasmids, circular extrachromosomal DNA molecules that can be transferred between bacterial strains and species. Therefore, effective methods to characterize bacterial plasmids and detect the presence of resistance genes can assist in managing AMR, for example, during outbreaks in hospitals. However, existing methods for plasmid analysis either provide limited information or are expensive and challenging to implement in low-resource settings. Herein, we present a simple assay based on CRISPR/Cas9 excision and DNA combing to detect antimicrobial resistance genes on bacterial plasmids. Cas9 recognizes the gene of interest and makes a double-stranded DNA cut, causing the circular plasmid to linearize. The change in plasmid configuration from circular to linear, and hence the presence of the AMR gene, is detected by stretching the plasmids on a glass surface and visualizing by fluorescence microscopy. This single-molecule imaging based assay is inexpensive, fast, and in addition to detecting the presence of AMR genes, it provides detailed information on the number and size of plasmids in the sample. We demonstrate the detection of several β-lactamase-encoding genes on plasmids isolated from clinical samples. Furthermore, we demonstrate that the assay can be performed using standard microbiology and clinical laboratory equipment, making it suitable for low-resource settings.

## Introduction

The growing incidence of antimicrobial resistance (AMR) has been classified as one of the main threats to global health^1,2^, and important measures need to be taken to limit the spread of genes conferring resistance to antibiotics^3^. Bacterial resistance genes are often carried by circular, extra-chromosomal DNA molecules called plasmids. Plasmids can range in size from one to several hundred kilobasepairs (kbp) and replicate independently of the chromosomal DNA. Many antibiotic resistance genes are located on plasmids, such as those encoding enzymes that break down the antibiotic molecules or pumps that expel antibiotics from the bacterial cell. Bacterial conjugation can transfer the plasmids from one cell to the other by direct cell-to-cell contact, thereby conferring antibiotic resistance to the receiving bacterium. Plasmids that can spread via conjugation are typically larger than ~ 50 kbp^4^. Importantly, one plasmid can harbor many different resistance genes, which means that a single conjugation event can make a bacterium that was previously susceptible to most antibiotics, multidrug-resistant. One key step in limiting the global spread of AMR is the rational use of antibiotics. This necessitates the development of novel techniques that can diagnose antimicrobial resistant infections, as well as confirm the spread of resistant bacteria in hospital settings so that proper antibiotic therapy can be prescribed, and appropriate infection prevention and control measures can be employed. Since the healthcare infrastructure, social hygiene practices and economic status of a country are directly correlated to the prevalence of AMR^5^, the diagnostic technique also needs to be fast, simple and cost-effective for favorable deployment in low-resource settings.

Despite their importance, methods for detailed characterization of plasmids are limited in the type of information they can reveal. In some methods, such as pulsed-field gel electrophoresis (PFGE) with S1 restriction, one can determine the number of plasmids in a sample and their size, but no information on the presence of specific (resistance) genes can be obtained^6^. PFGE could potentially be combined with southern blotting to target genes; however, it is then challenging to use in low-resource settings. Other methods, such as polymerase chain reaction (PCR) can detect a specific gene in a sample, but no further information on the plasmid composition can be obtained. Short-read next-generation sequencing with a typical read length of < 400 bp often fails to assemble full circular plasmid contigs due to the frequent presence of repeat regions larger than the read length, offering only limited information in the context of plasmids. To obtain complete information on the plasmid composition in a sample, the only existing general solution is long-read sequencing^7^, which requires extensive sample preparation and bioinformatics analysis.

We have previously reported a method for investigating single DNA molecules using nanofluidic channels and a labeling scheme based on optical DNA mapping (ODM), for detailed analysis of bacterial plasmids^8,9^. Using ODM, several important characteristics of plasmids can be obtained in a single experiment, including how many different plasmids there are in an isolate, their size, as well as a barcode that can be used for identification and tracing the spread of plasmids^10–12^. By adding a step where the circular plasmids are linearized with CRISPR/Cas9 excision, we can also determine on which plasmid a specific (resistance) gene is located^13^ which is important to track the potential spread of plasmids, for example during AMR outbreaks^14–16^.

In the current format of the ODM assay, the DNA molecules are stretched by confinement in nanochannels and imaged using advanced fluorescence microscopy, which limits its general use. An alternate strategy to image single DNA molecules is to stretch them on glass surfaces by a process called DNA combing^17–19^. This strategy offers an inexpensive alternative that does not require access to a nanofabrication facility. Several groups have reported different DNA combing methods and the parameters affecting the combing efficiency^20–23^. DNA combing has been demonstrated for stretching DNA on a megabase scale for fiber-FISH analysis^24^ and high-resolution optical mapping for bacterial genome analysis and species identification^25–27^. However, most reported combing strategies rely on DNA ends fraying to bind to the hydrophobic surface for combing to happen^28,29^ and can therefore not be used for stretching circular DNA molecules.

In this report, we present a simple “cut and stretch” assay based on CRISPR/Cas9 mediated targeting of AMR genes and linearization of circular plasmids^13^. When DNA is stretched on glass, the linearized plasmids appear twice as long compared to their circular form^30,31^, indicating the presence of the AMR gene. Our assay is inexpensive, fast, reliable and can find broad application in AMR gene detection. It can be performed on simple fluorescence microscopes that are present in many microbiology and clinical laboratories, even in low- and middle-income countries. The images can even be acquired by a smartphone camera mounted on the eyepiece of the microscope, lowering the critical barrier for adopting this new plasmid characterization method. We demonstrate the assay using a simple microscope that is identical to setups already present in many African countries via a tuberculosis diagnosis program^32–34^. Our method could replace S1-PFGE and complement PCR in plasmid analysis routines and has the potential to become a general tool in plasmid microbiology as well as in epidemiology and diagnostics.

## Results and discussion

**Figure 1:**
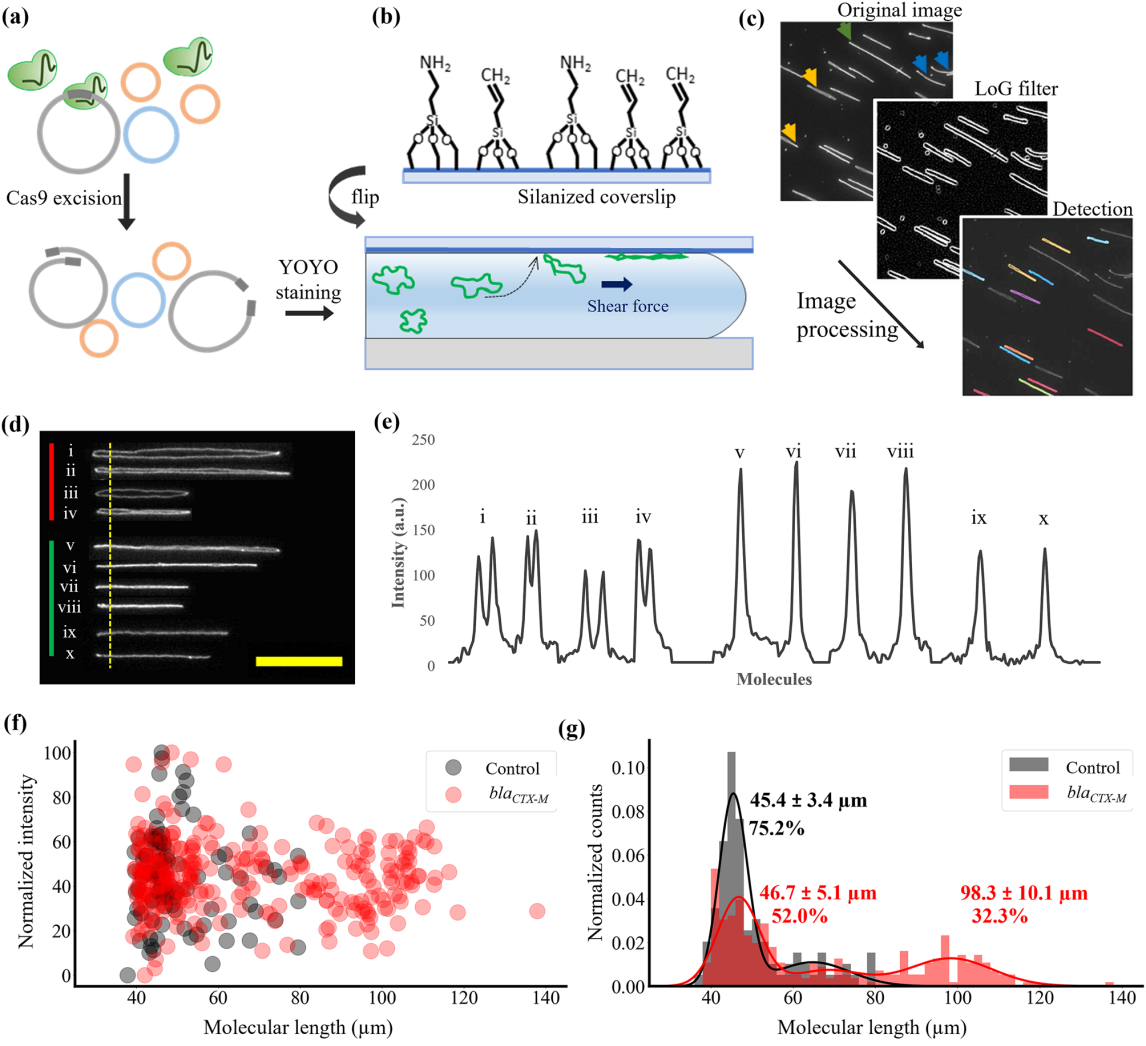
Experimental workflow. (**a**) Cas9-gRNA is added to the plasmid sample. If the AMR gene is present, Cas9 makes a double-stranded excision, resulting in plasmid linearization. (**b**) Glass coverslips are functionalized with a mixture of silanes with amine and vinyl terminal groups. DNA molecules are stained with YOYO-1 and stretched by capillary force between the silanized coverslip and a glass slide. (**c**) Image processing to detect molecules. A Laplacian of Gaussian (LoG) filter is used for edge detection and molecular length, width, eccentricity, and straightness filters are used to accept or reject the detected molecules. Examples of molecules not accepted are marked with yellow (too wide), blue (too curved) or green arrows (overlapping molecules), respectively. (**d**) Representative images of molecules observed during imaging. Molecules i-iv (red bar) are rejected and molecules v-x (green bar) are accepted. (**e**) Intensity profile for molecules in (**d**) along the dotted yellow line. Intensity is higher where two strands of the circular plasmid are localized together. Scale bar 15 µm. (**f**) Length vs. intensity plot for a sample carrying a single plasmid. Data for Cas9 targeting the bla_CTX-M_ gene is shown as red dots and control as black dots. (**g**) Length histograms of the data in (**f**).

We present herein an assay to determine the number of plasmids in a sample, their size and, importantly, on which plasmid a specific (resistance) gene is located, based on fluorescence microscopy of single plasmids stretched on functionalized glass coverslips. The fundamental principle behind the gene detection is that when fully stretched, a linear plasmid molecule will be twice the length and half the emission intensity per pixel of the corresponding circular configuration^30,31^. Hence, if Cas9 causes a double-stranded break at a gene of interest in a plasmid, this can be detected since the circular plasmid will be converted into its linear configuration^13^ (Fig. 1a). By comparing data between a control sample and a sample where Cas9 is targeting a gene of interest, it is possible to directly confirm if a plasmid has been linearized or not, and hence confirm the presence of that gene. We demonstrate detection of the two β-lactamase resistance gene groups *bla*_CTX-M_ group-1 and *bla*_NDM_ in different bacterial isolates of varying plasmid complexity. These genes encode for β-lactamase enzymes that inactivate β-lactam antibiotics (including the last-resort carbapenems in the case of *bla*_NDM_) and hence confer multidrug-resistance. The principle is however general, and any gene encoded on a plasmid can be identified by using the corresponding guide RNA (gRNA) for specific Cas9 cleavage. Most plasmids used here were extracted from *Escherichia coli* or *Klebsiella pneumoniae* isolated from urine, fecal or sputum samples from patients admitted to Siriraj Hospital in Bangkok, Thailand during 2018 and 2019.

### DNA combing, molecule detection and first proof-of-concept

To stretch the DNA, we functionalized glass coverslips with a 1% (v/v) mixture of Allyltrimethoxysaline (ATMS) and (3-Aminopropyl) trimethoxysilane (APTES) in acetone (see “Methods”)^35,36^. The functionalized coverslips were assembled on a microscope glass slide and YOYO-stained DNA was dispensed at the edge of the coverslip. The DNA solution was pulled between the two glass surfaces by capillary force and DNA molecules were instantly stretched on the silanized glass surface (Fig. 1b). Previously reported protocols for DNA combing typically use vertical dipping or moving droplet methods^17,29,37^, where DNA molecules attach to the hydrophobic glass surface and stretch because of the receding fluid meniscus. These methods have been exclusively used for combing linear DNA molecules and rely on the fraying of DNA ends (and a very narrow pH range, 5.5–6.0) to attach to the glass surface. After stretching, the DNA molecules are in the air phase and the samples are dried/baked (and sometimes rehydrated) before imaging^27^. In contrast, we did not use an air–liquid interface to stretch DNA and in our setup, combing took place entirely in the liquid phase. Surface modification with ATMS and APTES resulted in a mix of vinyl (hydrophobic) and amino (hydrophilic and positively charged) groups on the glass surface. When the liquid was pulled between the two glass surfaces, the DNA molecules attached to the amino groups on the top glass surface by electrostatic interaction. At the same time, the molecules also experienced a shear-induced deformation/stretching force^38^, which stretched the DNA in the direction of the fluid flow. The combination of the binding and stretching forces that DNA experienced at the solid–liquid interface resulted in DNA molecules partitioning onto the glass surface in an almost fully stretched conformation, where they were stabilized by multiple attachment points. Since our method does not rely on fraying DNA ends, it is more tolerant to solution pH and could be used to stretch both linear and circular DNA molecules under the same combing conditions. We typically used 3 µl of 0.12 µM DNA concentration to comb the DNA and it resulted in optimally spaced molecules for imaging and analysis. This corresponds to ~ 200 pg DNA for one stretching attempt. As the fluid flowed between the two glass surfaces and DNA molecules got deposited on the coverslip, a density gradient was established where the loading side had more molecules per unit area. During imaging, areas of the coverslip with sufficient molecule density and optimal spacing were imaged. A lower DNA concentration can be easily used, since a lower density of molecules on the surface only means more time is needed to image sufficient molecules to build up the statistics.

After combing, the stretched DNA molecules were imaged, and the images were processed with custom MATLAB routines, to extract molecule length and average intensity per pixel. We used the Laplacian of Gaussian (LoG) edge detection algorithm to detect molecules, followed by shape filtering based on length, width, eccentricity, and straightness (molecule-to-convex-hull ratio) of the detected molecules. Figure 1c shows the molecule detection workflow where some representative molecules typically rejected by the algorithm are marked with yellow (too wide), blue (too curved) or green arrows (overlapping molecules), respectively. Figure 1d shows representative images of stretched molecules. The molecules in the top panel (red bar, molecules i–iv) are circular molecules in a stretched elliptical configuration with two strands visible. In our experience, such configuration adds significant variation to the plasmid length and intensity per pixel data and such molecules were therefore rejected during shape filtering. The molecules in the lower panel (green bar, molecules v–viii) have the best possible configuration (rod-like shape) with the two strands of the circular molecules almost co-localized. Molecules satisfying such shape criteria were accepted and used for further analysis. Molecules *ix* and *x* are linear molecules. Figure 1e shows the intensity profile of molecules in Fig. 1d measured along the vertical dotted yellow line (single-pixel wide line). Note that for molecules *i–iv*, two peaks corresponding to two strands of stretched circular molecules were seen. For molecules *v–viii,* only one intensity peak was seen since the two strands of the circular plasmids were co-localized, and the intensity values were higher^30,31^ than for molecules *i–iv*. The intensities for molecules *ix* and *x* were similar to molecules *i–iv.* This difference in average intensity per pixel for the detected molecules was used to distinguish circular stretched (rod-like shaped) molecules from linear molecules of similar length.

As the first demonstration of our method, we selected a previously characterized sample with only one plasmid carrying the *bla*_CTX-M_ gene. This plasmid, designated as pUUH239.2, is a 220 kbp plasmid originally isolated from a clinical *K. pneumoniae* strain that caused a hospital outbreak in Uppsala, Sweden^39^. The plasmid preparation was treated with Cas9 using either a gRNA targeting the *bla*_CTX-M_ gene or a decoy gRNA (a gRNA targeting a human gene). After the Cas9 reaction, plasmids were stained and stretched on glass slides. As hypothesized, we observed longer molecules for the plasmid sample treated with Cas9 with *bla*_CTX-M_ gRNA compared to the control case with decoy gRNA (Fig. 1f). Length histograms (Fig. 1g) were fitted with multiple Gaussian curves to identify different populations in the data. Figure 1g shows that in the control case the dominant population (75.2%) was 45.4 ± 3.4 µm long. However, after plasmid digestion targeting *bla*_CTX-M_, the fraction of plasmids that were ~ 45 µm long was reduced from 75.2% to 52.0% and a new population (32.3%) emerged with a length of 98.3 ± 10.1 µm, which is close to twice the length of the original population. This result indicates that Cas9 targeting the *bla*_CTX-M_ gene linearized the circular plasmid and the change in length distribution could be used to infer the presence of the gene of interest. The fitting parameters for the length histograms in Fig. 1g are summarized in Supplementary Table ST1.

There is a possibility that two or more copies of an AMR gene are present on a plasmid. It is important to understand if a plasmid is carrying more than one copy of the gene as it may affect assay results and interpretation; however, from a clinical standpoint the more important question is if the gene is present or not, since the bacteria will become resistant irrespective of the number of copies of the gene. When multiple copies of the same gene are present on a plasmid, Cas9 will cut the plasmid at multiple locations and the length of the linearized molecules will be different from the predicted length. The change in length of the linearized plasmid due to multiple Cas9 cuts will show as a shift in the length histogram and the ability to detect two copies of gene on a plasmid will depend on how far apart on the plasmid they are located (see Supplementary Note 1).

### Extended-spectrum β-lactamase and carbapenemase gene detection in complex samples

**Figure 2:**
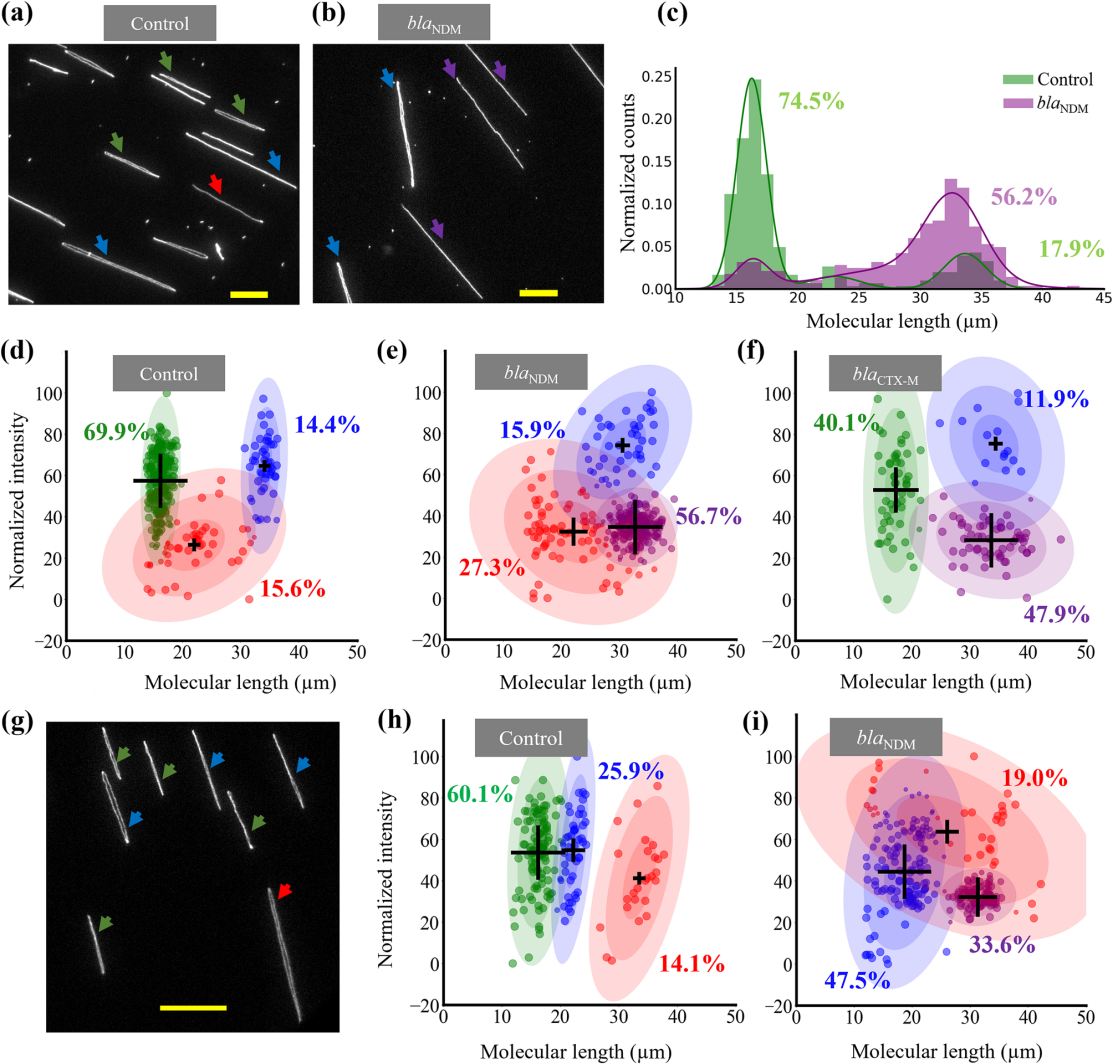
Extended-spectrum β-lactamase and carbapenemase genes in samples with multiple plasmids. Representative images of plasmids after control (**a**) and bla_NDM_ (**b**) reactions. In the control case, two circular populations (green and blue arrows) were observed, along with linear molecules (red arrow). After the bla_NDM_ reaction (**b**), two plasmid populations of similar lengths (blue and purple arrows), but different emission intensities, were observed. Scale bars 10 µm. (**c**) Length distribution of plasmids after control and bla_NDM_ reactions for the control sample (green histogram) and the bla_NDM_ reaction (purple histograms) (**d**–**f**) Scatter plots with GMM clustering after control (**d**), bla_NDM_ (**e**) and bla_CTX-M_ group 1 (**f**) reactions, respectively. (**g**–**i**) Carbapenemase gene detection in a sample with three plasmids. (**g**) Representative image showing plasmid composition of the sample. The three different sized plasmids are marked with green, blue and red arrows. All molecules in the image are circular. Scale bar 15 µm. (**h**–**i**) GMM clustering results for the three-plasmid sample after the control reaction (**h**) and after the bla_NDM_ reaction (**i**). The population clusters identified by the algorithm are presented in different colors in the scatter plots. The mean values are marked with the ‘ + ’ signs (scaled to the weights of the clusters). The percentage values represent the percentage of the population in that cluster. The colored ellipses around the clusters represent standard deviations of the mean. The sizes of the dots are scaled to the probability of belonging to that cluster.

For the second demonstration, we used our assay for two samples with multiple plasmids. Since the assay is based on single DNA molecule imaging, we can easily detect sub-populations in the sample, allowing us to determine the number of plasmids present in the sample and which plasmid that is carrying the AMR gene. This information is important from a microbiology and epidemiology perspective and can help in tracing the spread of plasmids that cause AMR. Another important aspect of plasmid analysis is to understand if two different (resistance) genes are located on the same plasmid. This is particularly relevant since if the two genes are located on the same plasmid they will conjugate together, meaning that bacteria that receive this plasmid will simultaneously acquire two genes, potentially encoding two different resistance mechanisms. By using Cas9 targeting two different genes of interest (extended-spectrum β-lactamase and carbapenemase genes in this case) it was possible to detect if they were on the same plasmid or not. Figures 2a–f show data for an *E. coli* sample with two plasmids (Fig. 2a) where we used Cas9 targeting either the *bla*_NDM_ gene family or the *bla*_CTX-M_ group 1 gene family. When the sample was digested with Cas9 targeting the *bla*_NDM_ gene, the smaller circular plasmids disappeared, and long linear molecules could be observed (purple arrows in Fig. 2b). As shown in Fig. 2c for the control case, two major peaks at 16.2 ± 1.2 µm (74.5% population) and 33.6 ± 1.7 µm (17.9% population) were observed. After the sample was digested with Cas9, the locations of the two peaks were 16.3 ± 1.3 µm (11.1% population) and 32.7 ± 2.3 µm (56.2% population). Although the location of the two peaks in the control and *bla*_NDM_ reactions were similar, the shift in the percentage of the population from one peak to the other indicated linearization of the smaller plasmid, as was also observed in the microscopy images. The interpretation of the results for this sample was non-intuitive since one plasmid was approximately twice the length of the other plasmid and when the smaller plasmid was linearized, we ended up with two populations of similar lengths, one linear and one circular. In Fig. 2d–f, we complemented the molecular extension data with the average emission intensity per pixel for each molecule. The rationale behind this is that circular DNA molecules are double-folded when they are stretched on glass, which means that the emission intensity per pixel is approximately twice that for a linear DNA molecule^30,31^. This can then be used to cluster the data in two dimensions, instead of one, which can help to distinguish between circular and linear plasmid populations showing comparable molecular extension. This is particularly helpful when plasmids of different lengths are present in a sample. The data points in the scatter plots were clustered using the Gaussian mixture model (GMM) employing the expectation–maximization algorithm.

As seen in Fig. 2d, we observed two major plasmid populations (at 69.9% and 14.4%) at high emission intensities per pixel and a third cluster (15.6%) made up of linear molecules from chromosomal DNA or plasmid shearing. After the *bla*_NDM_ reaction, the data points from the cluster corresponding to the smaller plasmid moved into a new cluster with a mean length of 32.7 ± 2.5 µm and representing 56.7% population (Fig. 2e). We then observed two clusters with mean lengths close to 32 µm, but with very different intensities per pixel, which confirmed that the smaller plasmid in the sample was carrying the *bla*_NDM_ gene and was cut at the gene site by Cas9 and linearized. When the plasmids were digested with Cas9 targeting the *bla*_CTX-M_ group 1 gene (Fig. 2f), the results were similar to the *bla*_NDM_ reaction. A fraction of the smaller plasmids was linearized and a new cluster at 33.7 ± 4.7 µm (47.9%) emerged. These results indicate that the smaller plasmid in this sample was carrying both the *bla*_NDM_ and *bla*_CTX-M_ group 1 genes. We also stretched linear λ-DNA (48.5 kilobase pairs) on glass (see Supplementary Note 2 and Fig. S2) and used it as a molecular ruler to estimate the size of the plasmids observed in the sample. The sizes of the two plasmids in this sample were estimated to be ~ 98 kbp and ~ 180 kbp, respectively. The presence of the *bla*_NDM_ gene in the plasmid preparation and plasmid size was validated using optical DNA mapping in nanochannels (see Supplementary Note 3 and Supplementary Fig. S6), a technique that has been extensively validated with long-read sequencing^13,14^. All fitting parameters and population percentages are summarized in Supplementary Table ST2.

We also wanted to explore the minimum number of data points needed in the length vs. intensity plots to classify the data into different clusters and interpret the results. It is difficult to propose a fixed number as it depends on relative sizes and abundances of different plasmid populations as well as on noise and spread of data. To understand how our results may be affected if data is sparse, we randomly sampled different fractions of the data in Fig. 2d and applied the GMM algorithm to cluster. We obtained similar data classification and cluster parameters even with 10% of the data or 30 total data points and interpretation of the result was not affected (see Supplementary Fig. S9).

Next, we demonstrate the detection of carbapenemase-encoding genes in a *K. pneumoniae* sample with three plasmids of different lengths (Fig. 2g). The lengths of three circular plasmids, as identified in the control sample (Fig. 2h), were 16.2 ± 1.8 µm, 22.2 ± 1.3 µm and 33.5 ± 2.9 µm, respectively, with 60% of data forming the cluster for the smallest plasmid. After the *bla*_NDM_ Cas9 reaction (Fig. 2i), we observed the emergence of a new cluster at 31.4 ± 2.2 µm carrying 33.6% of the molecules and a simultaneous reduction in the ~ 16 µm cluster that was observed in the control sample. The mean length for the new cluster was close to twice the mean length of the ~ 16 µm cluster, and the mean intensity per pixel was lower, which indicated linearization of plasmids and the presence of the *bla*_NDM_ gene on that plasmid. The sizes of the three plasmids in this sample, when converted to kilobase pairs, were ~ 95kbp, ~ 120 kbp, and ~ 180 kbp, respectively, and the *bla*_NDM_ gene was on the smallest plasmid. The presence of the *bla*_NDM_ gene in this plasmid preparation and the plasmid size was validated using optical DNA mapping in nanochannels (see Fig. S7). All fitting parameters and population percentages are summarized in Supplementary Table ST2.

When working with a sample with multiple plasmids, there is a possibility that two plasmids of similar lengths are present. In such a case, the ability of the method to distinguish between the two sub-populations is important. The minimum size difference detectable depends on the standard deviation or the spread of the population and in our assay if two sub-populations are three standard deviations of the mean apart from one another, they can be resolved (see Supplementary Note 4). If there are two plasmid populations with mean lengths less than three standard deviations of the mean apart and one of them carries an AMR gene, the two plasmids can be distinguished as one of them will get linearized by Cas9.

### Detection of carbapenemase genes

In the next example, we demonstrate that the assay can address the clinically important question if a plasmid sample carries a gene that produces an enzyme that degrades a specific type of antibiotic, but where the exact identity of the gene is not as important. In this case, it is possible to use a cocktail of gRNAs targeting a group of AMR genes and if the Cas9 reaction results in the linearization of a plasmid, the presence of that type of gene can be confirmed^40^. This approach can, for example, provide information to the healthcare staff about the predicted resistance and assist them in making quick clinical decisions, such as avoiding administering certain classes of antibiotics or isolating patients that carry pathogens with particularly problematic resistance. Once the AMR gene group is confirmed, it is possible to confirm the presence of a specific gene by separate Cas9 reactions. One group of enzymes that are of relevance in this context is carbapenemases, enzymes that degrade carbapenems that are often considered as a group of last-resort antibiotics. Clinically important carbapenemase genes include *bla*_NDM_*, bla*_OXA-48_, *bla*_KPC,_ *bla*_VIM_ and *bla*_IMP_.

**Figure 3:**
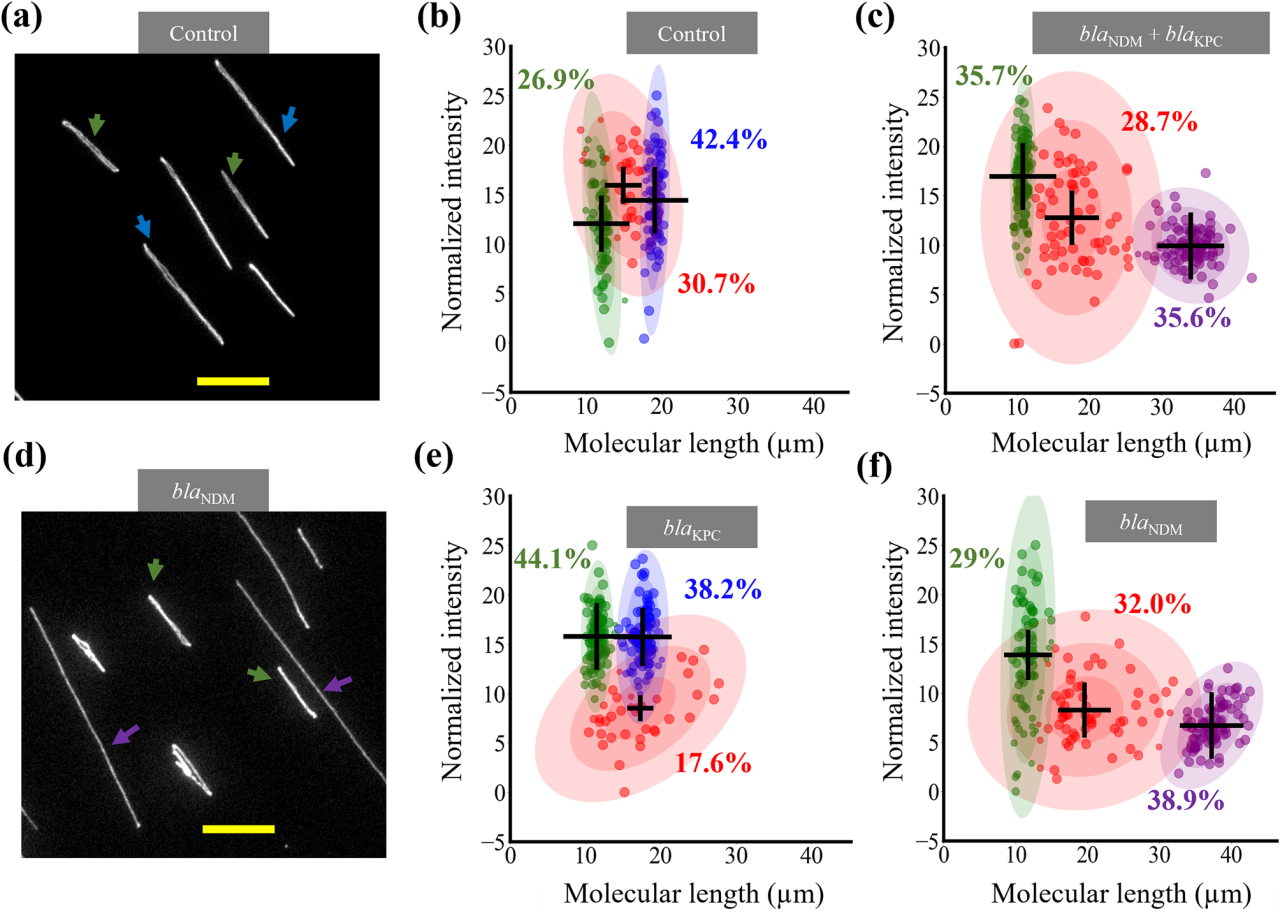
Carbapenemase gene detection in a sample with two plasmids. (**a**) Image of plasmid sample after the control reaction. The two plasmids observed are marked with green and blue arrows, respectively. (**b**,**c**) Length and intensity per pixel after the control reaction (**b**) and the carbapenemase gRNA cocktail reaction (**c**) clustered using GMM. (**d**) Image of plasmid sample after the bla_NDM_ reaction. The larger plasmid in (**a**) was not observed. The smaller plasmid is marked with green arrows and the linearized larger plasmid is marked with purple arrows. (**e**,**f**) Data clustered after separate bla_KPC_ (**e**) and bla_NDM_ (**f**) reactions. The population clusters identified by the algorithm are presented in different colors. The mean values are marked with ‘ + ’ signs (scaled to the weights of the clusters). The percentage values represent the percentage of the population in that cluster. The colored ellipses represent the standard deviations of the mean. Scale bars 10 µm.

We demonstrate the detection of carbapenemase-encoding genes in a plasmid preparation from *E. coli* (Fig. 3), where we targeted *bla*_NDM_ and *bla*_KPC_ genes. Figure 3a shows the two plasmids in the sample in their circular configuration. In the control sample, we observed two major populations of plasmids at lengths of 12.3 ± 0.3 µm and 19.0 ± 0.7 µm representing 26.9% and 42.4% of the total population (Fig. 3b). The cluster at 13.8 ± 2.8 µm (marked in red) identified by the clustering algorithm can be attributed to subsets of the main plasmid populations stretching non-uniformly. When the sample was treated with a Cas9-gRNA cocktail targeting the two carbapenemase-producing genes (*bla*_NDM_ and *bla*_KPC_), the longer plasmid was linearized and a new cluster was observed at 33.9 ± 2.7 µm (35.6% of the total population), confirming that this plasmid carried a carbapenemase-encoding gene (Fig. 3c). The length for the new cluster was close to double the length for the cluster at ~ 19 µm in the control sample. The appearance of the new cluster was also accompanied by thinning of the corresponding previous cluster (after the Cas9 reaction we observed a very broad distribution with a mean of 17.5 µm representing 28.7% of the population). We then set up two separate Cas9 reactions, using gRNAs targeting either the *bla*_NDM_ or the *bla*_KPC_ gene. The larger plasmids seen in the control case disappeared when using Cas9 targeting *bla*_NDM_ and instead long linearized molecules were observed (Fig. 3d). As can be seen in Fig. 3e–f, the clustering and population distribution for the *bla*_KPC_ reaction were similar to the control case and those for *bla*_NDM_ were similar to when the plasmids were linearized. From this, we inferred that the carbapenemase-encoding gene in the sample was *bla*_NDM_, and not *bla*_KPC,_ and that it was present on the larger plasmid. The presence of the *bla*_NDM_ gene in the larger plasmid was confirmed using optical DNA mapping in nanochannels (see Fig. S8). All fitting parameters and population percentages are summarized in Supplementary Table ST3.

### AMR gene detection with a simple microscope and smartphone imaging

**Figure 4:**
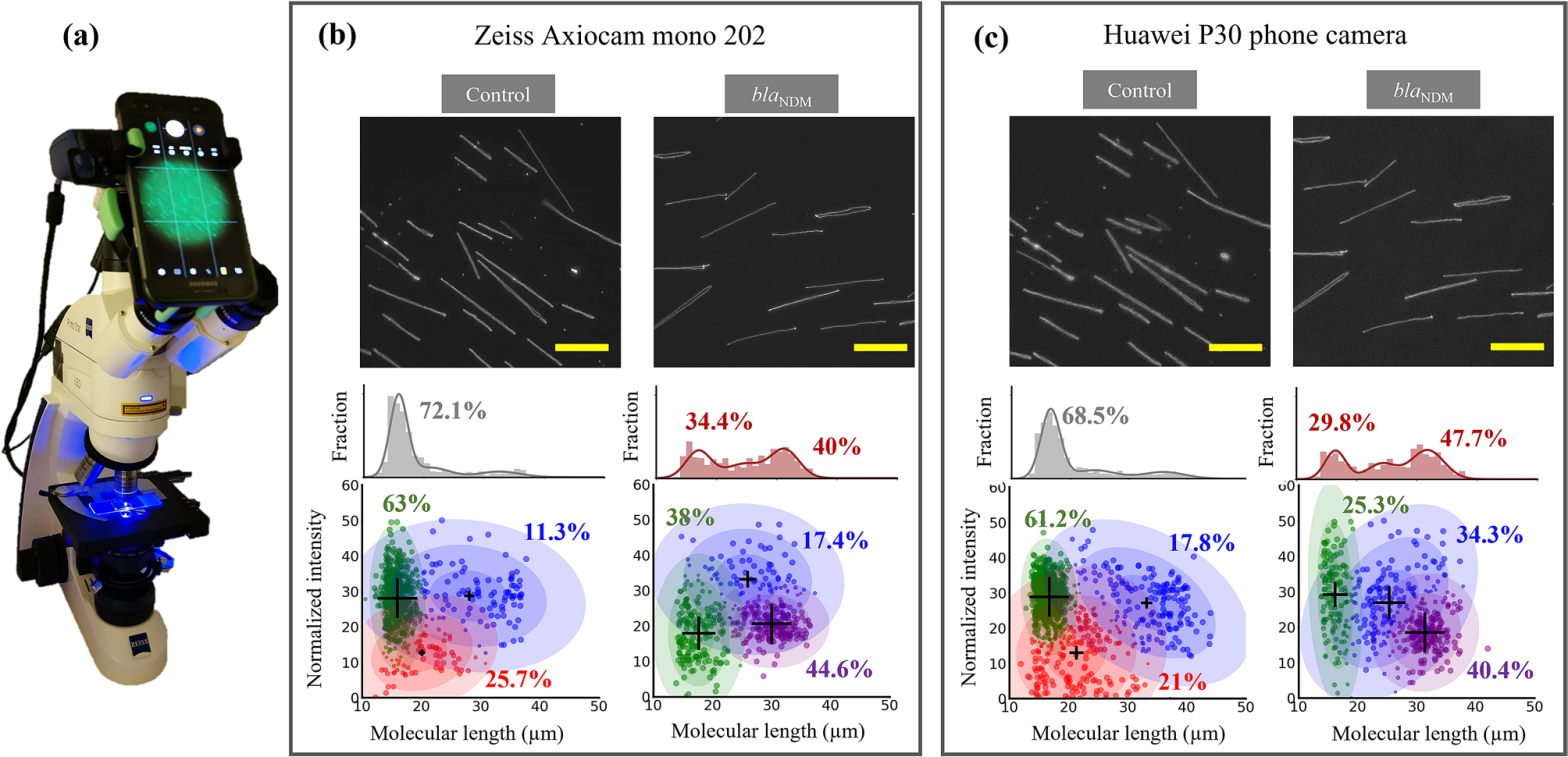
Use of a simple fluorescence microscope and smartphone camera for carbapenemase gene detection. (**a**) Zeiss PrimoStar iLED microscope equipped with Axiocam mono-202 and Huawei P30 smartphone camera was used to image plasmids stretched on glass. (**b**) DNA images for the control and bla_NDM_ reactions captured using the Axiocam mono-202 camera (top) and the corresponding length histograms (middle) and cluster plots (bottom). (**c**) DNA images and data analysis for images acquired for the sample using the Huawei P30 smartphone camera. Images shown are acquired from the same location on the coverslip. The population clusters identified by the algorithm are presented in different colors. The mean values are marked with ‘ + ’ signs (scaled to the weights of the clusters). The percentage values represent the percentage of the population in that cluster. The colored ellipses represent the standard deviations of the mean. Scale bars:20 µm.

All experiments so far were performed on a high-end fluorescence microscope (Zeiss, AxioObserver.Z1) equipped with a scientific-CMOS camera (Photometrics Prime 95B), a 100 × α Plan-Apochromat oil objective (Zeiss, NA = 1.46) and an LED illumination source (Colibri, Zeiss). To make the method applicable for broad use, it is important to demonstrate that it can also be used on simple fluorescence microscopes. We decided to use a Zeiss Primo-Star iLED microscope (Fig. 4), equipped with a Zeiss Axiocam mono-202 CMOS camera, a 100 × Plan-Achromat oil objective (Zeiss, NA = 1.25) and a 455 nm LED illumination source. One important reason for using this specific microscope is that it is used in low- and middle-income countries for diagnosing tuberculosis^32–34^. This means that our method can be directly transferred to such a setting for simple genetic analysis of plasmids. We also coupled a smartphone camera (Huawei P30 main camera, 40 megapixels) to the microscope eyepiece (using Carson HookUpz 2.0 Optics Adapter for Smartphone) and imaged the stretched DNA using the smartphone camera to evaluate if comparable results could be obtained with smartphone imaging which would make the method even more general.

Figure 4 shows images and data for a plasmid preparation from an *E. coli* isolate after the control and *bla*_NDM_ reactions, taken using the Axiocam mono-202 camera and the Huawei P30 camera on the PrimoStar microscope, respectively. The images shown in Fig. 4 were taken from the same location on the coverslip for direct comparison between the two cameras. The images were captured in .tiff format on the Axiocam mono-202 (default mode) and .jpeg format with the smartphone. Although Huawei P30 offers the possibility of image acquisition in the .RAW format, we wanted to work with .jpeg images to demonstrate that any smartphone that can capture .jpeg images can be used for data acquisition.

The DNA sample used for this experiment was the same sample as in Fig. 2a–f and the sample contained two plasmids. Imaging the same sample provided the possibility of comparing assay performance between the high-end AxioObserver.Z1 and the simple PrimoStar microscope. One very clear difference compared to AxioObserver.Z1 was that the images on PrimoStar suffered from non-uniform illumination across the field of view and appear curvilinear (pincushion distortion, typical of wide-angle lenses) when the smartphone camera was used. This resulted in more noise in the length and intensity per pixel data for DNA molecules compared to the data from AxioObserver.Z1 (Fig. 2a–f). DNA molecules in the images were detected as described earlier and the extracted data was used to create the histograms and scatter plots in Fig. 4. For the control case, a dominant population was seen at around 16 µm and after the Cas9-*bla*_NDM_ reaction, this population thinned and a new population at around 32 µm emerged (see length histograms). A similar shift in population distribution was detected with images acquired from Axiocam mono-202 and Huawei P30 cameras. The data was clustered using Gaussian mixture models and the number of clusters, the cluster parameters, and the shifts in population distributions were similar for the two cameras and also similar to the data acquired using AxioObserver.Z1 (for more discussion on data presentation, see Fig. 2a–f). These results demonstrate that even a simple fluorescence microscope equipped with a smartphone camera can be used to detect the presence of the *bla*_NDM_ gene on the plasmid. Moreover, plasmid linearization is evident in the images, which makes detection of AMR genes possible even “by eye”. This completely eliminates the need for cameras, allowing even simpler microscopes to be used for simple plasmid genetics.

### Plasmid length validation

**Figure 5:**
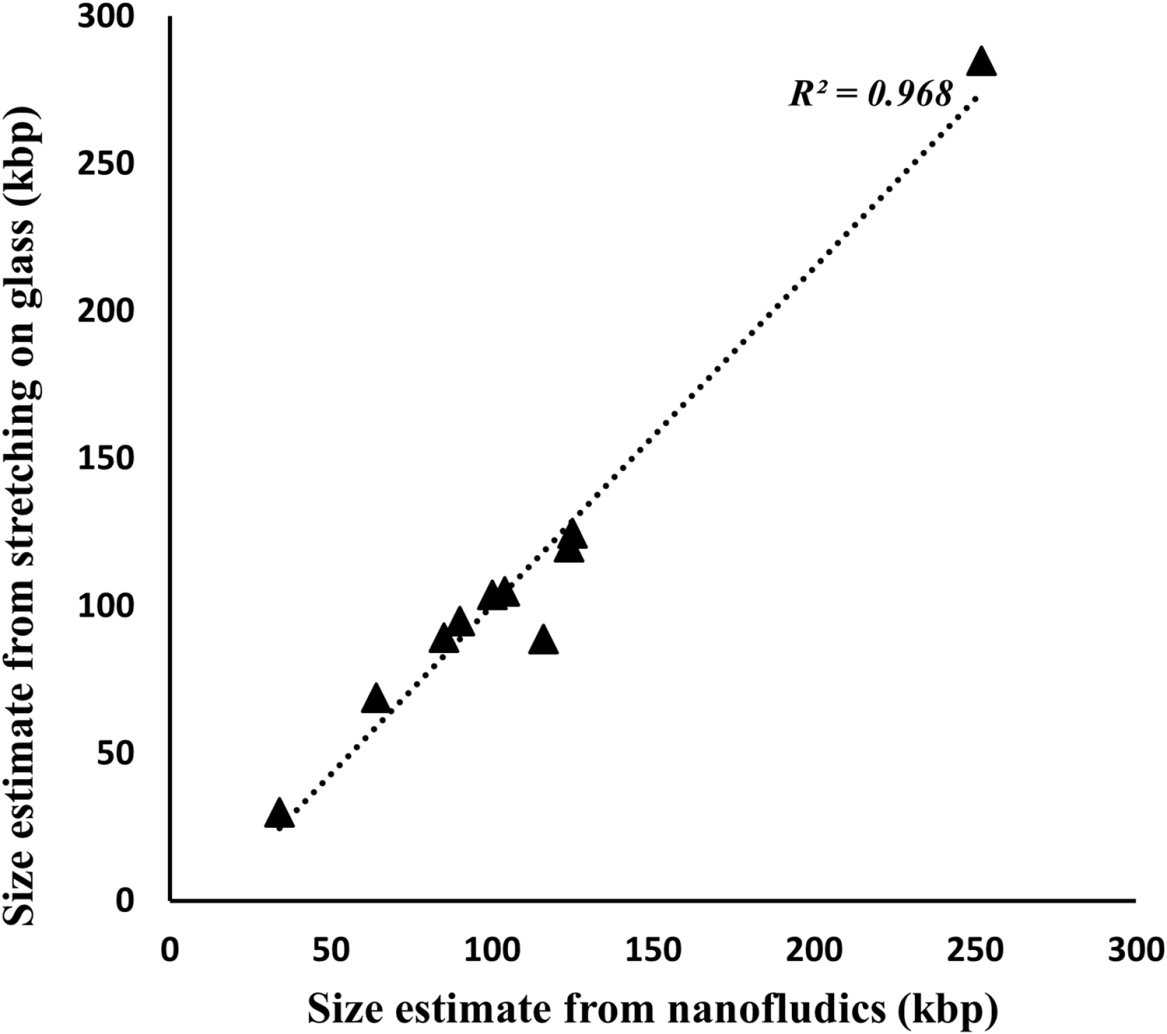
Size estimate comparison between DNA stretching on glass and in nanochannels for ten plasmids, ranging in size from 30 to 250 kbp, plotted and fitted with a straight line.

To validate the estimated size of the plasmids by DNA combing, we compared the length data between DNA combing and stretching in nanochannels. Since confining DNA in nanochannels for plasmid analysis is a very established assay in our laboratory and can give an accurate estimate of DNA length^30^, we wanted to evaluate if the length data from DNA combing matched the data from nanochannels. In Fig. 5, data for ten plasmids (including the plasmids used in the demonstrations in Figs. 1, 2, 3, 4) ranging in size from ~ 30 kbp to ~ 250 kbp were plotted and fitted with a straight line. We obtained a coefficient of determination (R^2^) value of 0.968, suggesting a high concordance in the size of plasmids measured using the two techniques, and verifying the size measured by combing.

## Conclusions

We demonstrate how the change in plasmid configuration, from circular to linear, when excised with Cas9 targeting a specific gene and combed on functionalized glass, can be used to detect the presence of that gene encoded on a specific plasmid. The assay also determines the number and sizes of plasmids in a sample. The plasmid size measurement was in very good agreement with the size estimate obtained from confining DNA in nanochannels. The assay can be carried out with simple equipment, using microscopy setups available in most laboratories. The ease and simplicity of the assay means that it can potentially be developed into a tool for plasmid microbiology, epidemiology, and AMR gene detection, particularly in low-resource settings.

## Methods

### Plasmid preparation

Bacterial samples were isolated from urine, feces or sputum of the patients admitted to Siriraj Hospital, Bangkok, Thailand in 2018 and 2019. The plasmid extraction was done with the plasmid purification kit NucleoBond^®^ Xtra Midi (Macherey–Nagel). Each isolate was cultured overnight in 100 ml low salt LB media (Sigma-Aldrich) and pelleted with centrifugation, 5000 rcf for 10 min at 4 °C. Next, the pellet was resuspended in resuspension buffer and further lysed and purified on columns according to Macherey–Nagel’s instructions. Precipitation of the plasmid DNA was done with isopropanol and then washed with ethanol (70%). Next, the sample was dried at room temperature and reconstituted in 50 μl TE-buffer. Nanodrop was used to determine the DNA concentration.

### Sample preparation/CRISPR-Cas9 reaction

The first step of the CRISPR-Cas9 reaction was the creation of a sequence-specific guide-RNA (gRNA). The 20-nucleotide CRISPR-RNA (crRNA) corresponding to the *bla*_NDM_, *bla*_KPC_ and *bla*_CTX-M_ group 1 genes and a decoy crRNA (control, with no potential target sites on bacterial plasmids) were designed and synthesized via Dharmacon (Horizon Discovery Ltd.). The target sequences of crRNAs for different genes were:

*bla*_NDM_: 5′ CCGCTGCATTGATGCTGAGC 3′

*bla*_KPC_: 5′ CAACCACCGCATCCGCGCGG 3′

*bla*_CTX-M_: 5′ CCGTCGCGATGTATTAGCGT 3′

decoy: 5′ GGTCCTTGTAACCATCGGTG 3′

The gRNA was created by mixing 0.5 nmol crRNA and 0.5 nmol trans-activating CRISPR RNA (tracrRNA, Dharmacon) in 1 × CutSmart^®^ Buffer (50 mM Potassium Acetate, 20 mM Tris–acetate, 10 mM Magnesium Acetate, 100 µg/ml BSA, pH 7.9 at 25 °C, New England Biolabs) into a 0.5 ml Eppendorf tube. The final volume was adjusted with milli-Q water to 15 µl. The mixture was incubated for 30 min at 4 °C. Next, 10 µM (0.05 nmol) gRNA, 600 ng Cas9 protein (Sigma-Aldrich), and 1 × CutSmart^®^ Buffer were added to a new Eppendorf tube and pre-incubated for 10 min at room temperature (25 °C). Finally, 60 ng DNA of plasmid sample and milli-Q water were added to the mixture to a final volume of 15 µl before incubation for 15 min at 37 °C.

### Activation of glass slides

Molecular combing was done on functionalized microscope cover glasses (Uncharged Cover Glass, 22 × 22 mm, Marienfeld). The cover glasses were incubated in a petri dish with a mixture of Allyltrimethoxysilane (ATMS, 95%, Sigma-Aldrich), (3-aminopropyl) triethoxysilane (APTES, ≥ 98%, Sigma-Aldrich) and Acetone (Sigma-Aldrich) in a 1:1:100 ratio for at least 30 min at room temperature. Next, the coverslips were cleaned with acetone and MilliQ water before drying with nitrogen. A functionalized coverslip was placed onto a Menzel-Gläser (Cut edges, frosted end, 76 × 26 mm, Thermo Scientific) and 3.7 µl of the DNA sample was pipetted onto the edge of the coverslip. The capillary force between the Menzel-Gläser and the treated coverslip pulled the solution under and the DNA was stretched and aligned on the surface of the glass. The edges were sealed with nail polish before placing onto the fluorescence microscope.

### YOYO-1 staining

The DNA was stained with 4.8 µM YOYO-1 (YOYO, Invitrogen) for visualization and diluted with 0.5 × TBE (Tris–Borate-EDTA, Medicago, diluted with mill-Q water from 10 × tablets) to a DNA concentration of 0.7 µM. Next, the mixture was incubated for 5 min at room temperature before dilution with 0.5 × TBE to a final DNA concentration of 0.13 µM. To reduce the risk of photo-nicking, β-mercaptoethanol (BME, Sigma-Aldrich) at 1% (v/v) was added to the mixture.

### Microscopy settings

The DNA molecules were visualized with a fluorescence microscope (Zeiss, AxioObserver.Z1) equipped with a scientific-CMOS camera (Photometrics Prime 95B), an LED light source (Colibri, Zeiss) and a 100 × oil immersion objective (Zeiss, NA = 1.46). The wavelength of the light used to excite YOYO was 475 nm and the emission filter was a fluorescein isothiocyanate (FITC) filter.

A cost-efficient and easy-to-use fluorescence microscope (Zeiss, Primo Star iLED) that is available in low- and middle-income countries was used to explore the adoption of the method to low resource settings. The microscope is equipped with a 100 × Plan-Achromat oil objective (Zeiss, NA = 1.25) and a single wavelength LED fluorescence illuminator with a 455 nm wavelength that was used to excite YOYO. It is also equipped with a 2 megapixels Zeiss Axiocam mono-202 camera.

### Smartphone camera imaging

The main camera of the Huawei P30 phone (camera model ELE-L29) was used at 1 × magnification for image acquisition. The images were acquired as .jpeg images in manual mode using the following settings – F-stop: f/1.8, Exposure time: ¼ seconds, ISO speed: ISO-4000. The camera was mounted on the microscope eyepiece using Carson HookUpz 2.0 Optics Adapter for Smartphone (Article no.: 301791001001, available through www.skogma.se).

### Data analysis

Custom MATLAB routines were used to segment the microscopy images. The segmentation software was based on applying a Laplacian-of-Gaussian (LoG) filter to each image. The width (scale parameter), σ, of the kernel of the LoG filter was set equal to the standard deviation, σ_PSF_, of the system’s optical point spread function. After thresholding the LoG-filtered image at 0 (using MATLAB’s built-in function imbinarize), we were left with a black-and-white image, where ideally a white region (a connected component of white pixels) corresponded to points along a DNA molecule. To remove false-positive regions, some post-processing steps were applied. First, for each white region, an edge score was calculated. The edge score was calculated by following the normal direction for the edge point and summing up the intensities in the inward and outward directions over a distance ω (where ω = 2.5σ_PSF_). The edge score was then the difference of intensities in the inward and outward directions. Hence, a large jump in intensity across the boundary gave a large edge score. Finally, the total edge score for a region was obtained by summing up the edge scores for all boundary points of that region. A threshold for the total edge score was set by MATLAB’s graythresh method. In the second post-processing step, we filtered out molecules based on length (major axis length), width (minor axis length), eccentricity and molecule-to-convex-hull ratio. Molecule-to-convex-hull statistic is the area of the “filled area” (signal) divided by the area of the smallest convex hull that can be fitted around the molecule. This was done using the regionprops method and dividing the property “FilledArea” by the property “ConvexArea” in MATLAB. The typical edge detection threshold and filter settings used were: threshold = 4.54e−05, width = 1–25 pixels, length = 160–Inf pixels, minimum eccentricity = 0.5–0.7, minimum molecule-to-convex-hull ratio = 0.5–0.7. The parameters varied slightly from sample to sample but the same parameters were used for all images of a sample. Through these postprocessing steps, unstretched or fragmented DNA molecules could, to some extent, be excluded. Once all molecules were identified, the length of each molecule was calculated in pixels and the mean intensity value for each pixel was measured along the stretched molecule. The intensity values were then averaged to give a mean intensity per pixel for every molecule. Molecule length in pixels was converted to micrometers by multiplying with the pixel size for images from different cameras. To account for experimental differences in intensities between samples, the mean intensities per pixel were normalized and scaled for data collected for each sample.

The output data after segmentation was analyzed using unsupervised clustering using the Gaussian Mixture Model (GMM) implemented in Python. The GMM clustering is a soft clustering approach without definitive boundaries between clusters. The clustering was performed in two dimensions where the first dimension corresponded to molecule length, and the second dimension corresponded to the mean intensity per pixel. The data was clustered in the number of clusters corresponding to minimum Bayesian information using the Bayesian Information Criterion (BIC). Each data point (defined by the tuple (length, mean intensity per pixel)) was assigned a probability of membership of a cluster. Each data point was assigned to the cluster with the highest probability and the sizes of plotted data points were scaled to visualize the probability of belonging to that cluster. This means that data points at the core of the cluster were visually larger than the data points at the interface of the two clusters. For each cluster, the mean value was marked with a ‘ + ’ sign and scaled to the relative weight of the cluster in the population.

### Ethical declarations

Sample collection and study design were approved by the Institutional Review Board of the Faculty of Medicine Siriraj Hospital, Mahidol University, Bangkok, Thailand (Si 571/2015) and all experiments were carried out as per prescribed guidelines and regulations of relevant institutions. All subjects who provided clinical specimens have signed the informed consent form at Siriraj Hospital, Mahidol University, Bangkok, Thailand.

## Supplementary Information

Supplementary Information.

## Data Availability

The imaging data and analysis routines used for molecule detection are available and can be obtained by making a reasonable request to the corresponding author.
