# Supplementary material for: A simple cut and stretch assay to detect antimicrobial resistance genes on bacterial plasmids by single-molecule fluorescence microscopy
Source: Sci Rep. 2022 Jun 3;12:9301. doi: 10.1038/s41598-022-13315-w (PMC9166776; doi:10.1038/s41598-022-13315-w)
Supplement: Supplementary file 1 — Supplementary Information. [file 41598_2022_13315_MOESM1_ESM.docx]

Supplementary information

For

**A simple cut and stretch assay to detect antimicrobial resistance genes on bacterial plasmids by single-molecule fluorescence microscopy**

Gaurav Goyal^1#^, Elina Ekedahl^1^^#^, My Nyblom^1^, Jens Krog^2^, Erik Fröbrant^2^, Magnus Brander^2^, Tsegaye Sewunet^3^, Teerawit Tangkoskul^4^, Christian G. Giske^3,5^, Linus Sandegren^6^, Visanu Thamlikitkul^4^, Tobias Ambjörnsson^2^, Fredrik Westerlund^1*^

^1^ Department of Biology and Biological Engineering, Chalmers University of Technology, Gothenburg, Sweden

^2^ Department of Astronomy and Theoretical Physics, Lund University, Lund, Sweden

^3^ Department of Laboratory Medicine, Karolinska Institute, Stockholm, Sweden

^4^ Faculty of Medicine Siriraj Hospital, Mahidol University, Bangkok, Thailand

^5^ Department of Clinical Microbiology, Karolinska University Hospital, Stockholm, Sweden

^6^ Department of Medical Biochemistry and Microbiology, Uppsala University, Uppsala, Sweden

^#^ These authors contributed equally to this work

^*^ To whom correspondence should be addressed: Email: [fredrikw@chalmers.se](mailto:fredrikw@chalmers.se)

**Supplementary Note 1: Two copies of the same gene on a plasmid**

In our assay we attempt to determine if a gene is present on the plasmid or not. It is interesting to explore if two copies of an AMR gene are present on the same plasmid; however, from a clinical perspective the more important question is if the gene is there or not. The bacteria will be resistant to a drug with a single or multiple copies of the AMR gene. However, multiple copies of the gene may affect the assay results and interpretation.

If two copies of a gene are present, in our assay, the plasmid will be cut at two locations. The ability to determine if two copies of the gene are present will depend on the distance between the two copies on the plasmid. Two cuts will result in shorter than the expected length of the linearized plasmids and a shift in mean value of population distribution that can be detected in data analysis.

Below we show how much shift in the mean value of the distribution is a significant shift. This can help us to expect as to what extent the shortening in length of linearized plasmid (due to two copies of a gene) could be detected in our assay. For the simulation, a random normal distribution of 300 datapoints was created with a mean value of 20 µm and a standard deviation of 4.5 µm (original population). A standard deviation of 4.5 µm is on the higher end of typical standard deviations observed in our experimental data. Then multiple similar distributions (test populations) were created with the same standard deviations but mean values displaced from the original population. Figure S1 below shows the p-values obtained from the t-test plotted against the distance between the means of the original and test populations. The results show that a shift > 0.5 µm in the mean value of the distribution is a significant shift. This corresponds to ~ 1.5 kbp for linearized plasmid in our assay. However, this is a theoretical estimate and depends on the standard deviation in the distribution. Based on these results we expect if two copies of the same gene are present on a plasmid and the plasmid is cut at two locations, our assay should would suggest shortening of the linearized plasmid if the two gene are atleast 5 kbp apart. However, it cannot be conclusive and the presence ot two copies of a gene would need to be validated by an orthogonal method.


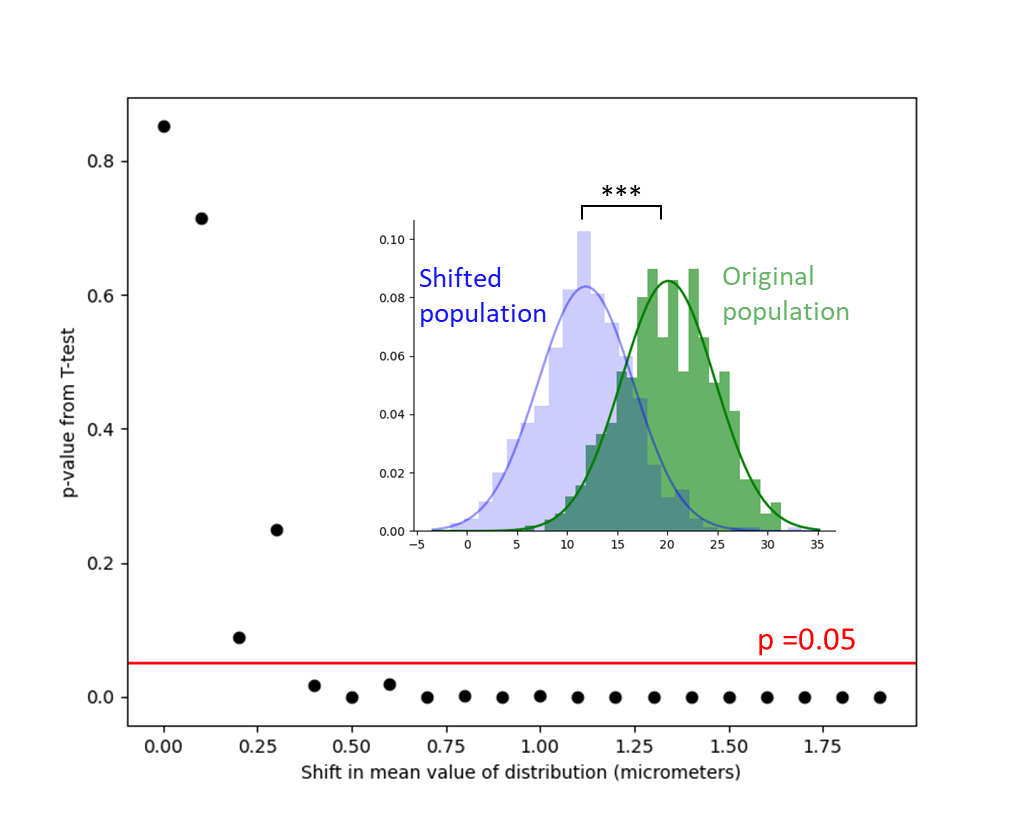


*Figure S1: p-values from t-test plotted against the distance between the means of the two distributions. Inset shows original population and a shifted population. The two populations means are significantly different.*

**Supplementary Note 2: Calculation of stretch factor**

For the determination of the length of the DNA stretched on the glass coverslips, lambda DNA was used to calculate a stretch factor. The linear lambda DNA molecule has a known size of 48.5 kbp and is thereby used as a size reference. In this experiment, YOYO-stained lambda DNA was stretched on different glass coverslips at six different time points where the average length of the DNA molecule was measured (Fig. S2). The average length of the lambda DNA was measured to be 16.1 µm which means that 1 µm is approximately 3 kbp. Plasmid DNA samples were stretched under the same experimental conditions and the stretch factor calculated from lambda stretching was used to estimate the size of plasmid DNA in kbp.

*Figure S2. Average stretching of lambda DNA on silanized coverslips. Each time point shows the mean and standard deviation of the mean.*

**Supplementary Note 3: Competitive-binding of YOYO/netropsin for optical mapping of DNA in nanochannels to detect the gene of interest**

For competitive-binding assay^1^, DNA is stained with fluorescent dye YOYO and non-fluorescent antibiotic molecule netropsin. YOYO binds to DNA indiscriminately; however, netropsin specifically binds to AT-rich regions. This results in netropsin outcompeting YOYO at AT-rich regions making DNA appear darker compared to the GC-rich regions (bound by YOYO) when imaged. When DNA molecules are stained with YOYO/netropsin and stretched and imaged in nanochannels, we obtain an intensity profile corresponding to the sequence of the DNA.

The use of optical DNA mapping combined with Cas9 excision to detect antibiotic resistance genes on bacterial plasmids is a very established assay in our laboratory and our results have been cross-validated by more conventional methods like PCR, NGS or pulsed-field gel electrophoresis ^2-5^. Briefly, when plasmids are cut with Cas9 targeting a gene of interest and then the optical mapping is performed on them, a significant fraction of the population is expected to be cut at a specific locus if the target gene is present. Since in optical mapping we can get the sequence-correlated intensity profiles of the molecules, consensus cutting of the plasmids can be detected by aligning the intensity profiles. If a consensus cut is present, the presence of the target gene can be inferred.

The detection of antibiotic resistance genes on plasmids demonstrated in Fig. 2-4 in the main text was validated by optical DNA mapping data shown in supplementary figures Fig. S6-S8.

**Supplementary Note 4: Resolution of the assay - minimum detectable size difference**

To answer the question of the resolution of the assay, we created a two-dimensional random normal distribution with mean value of 20 and a standard deviation of 2.5 (original population). This is very similar to population distributions in the experimental data. Further, multiple similar distributions (test populations) were created with the same standard deviations but mean values shifted from the original population (Figure S3).


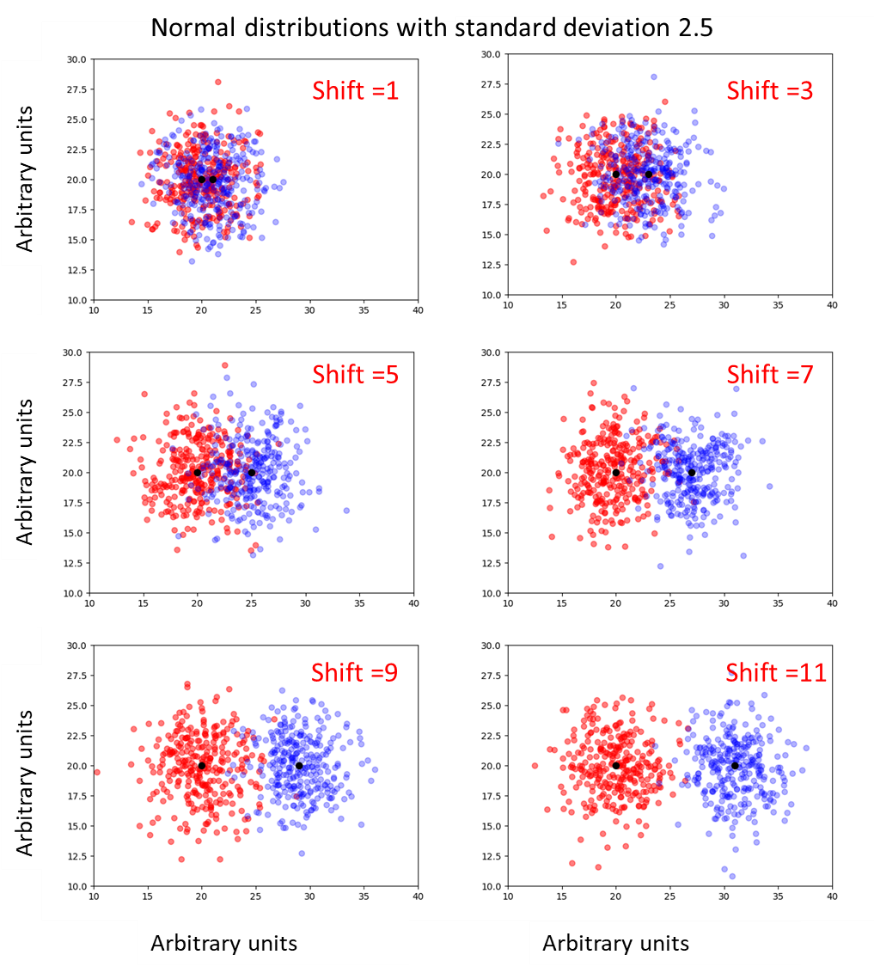


*Figure S3: Normal distributions with standard deviations 2.5 and different distance between the means. The means of the populations are marked with back dots.*

Then we applied the Gaussian Mixture Model algorithm to sets of two populations – original population and one of the shifted populations. The motivation was to understand –

1. If there are two sub-populations with means very close to each other, will they be classified as one population or two populations?
2. What should be the minimum distance between the means for these sub-populations to be classified as two populations?

Using the Bayesian information criteria it was determined if the data can be classified in one or two sub-populations. The minimum distance required for the data to be classified into two sub-populations is dependent on the standard deviations (spread) of the populations and the simulation was repeated for different distances between means and different standard deviations. The results in Fig R4 indicate that a minimum of 6 units distance is required at standard deviations of 2.5 and minimum of 11 units distance is required for standard deviations of 4.5 for the data to be classified into two clusters.


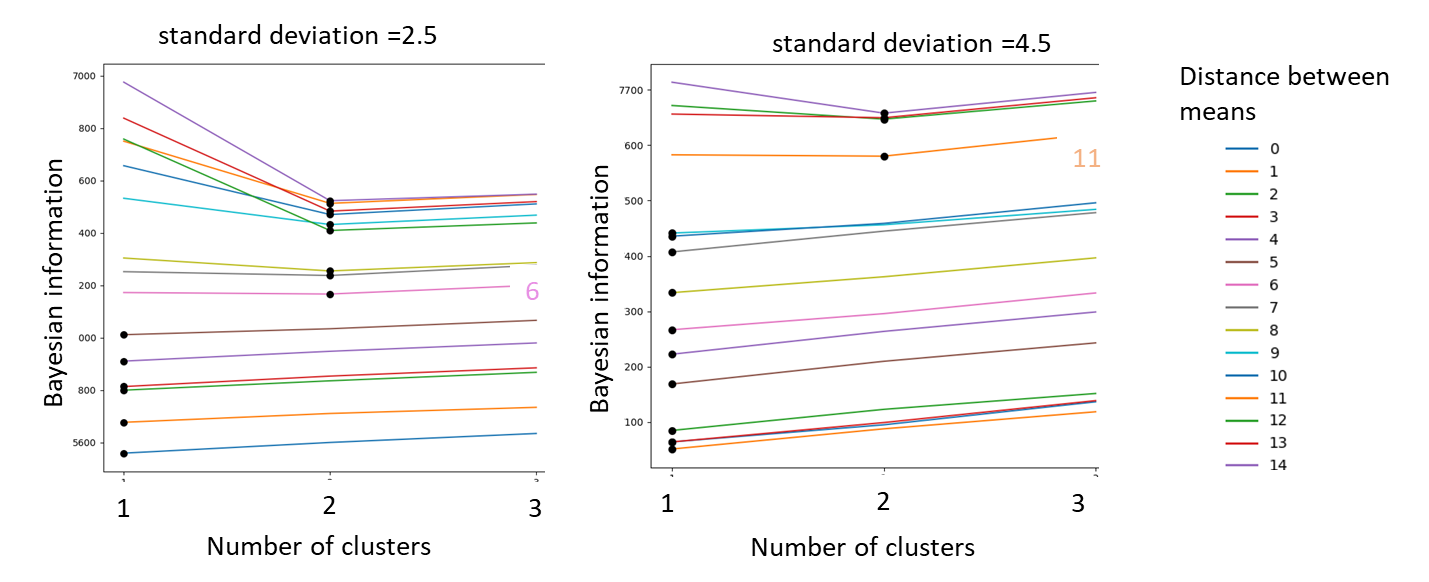


*Figure S4: Bayesian information criteria to determine the number of clusters in population. Plots show the number of clusters identified for different standard deviations and different distance between means of the two simulated populations.*


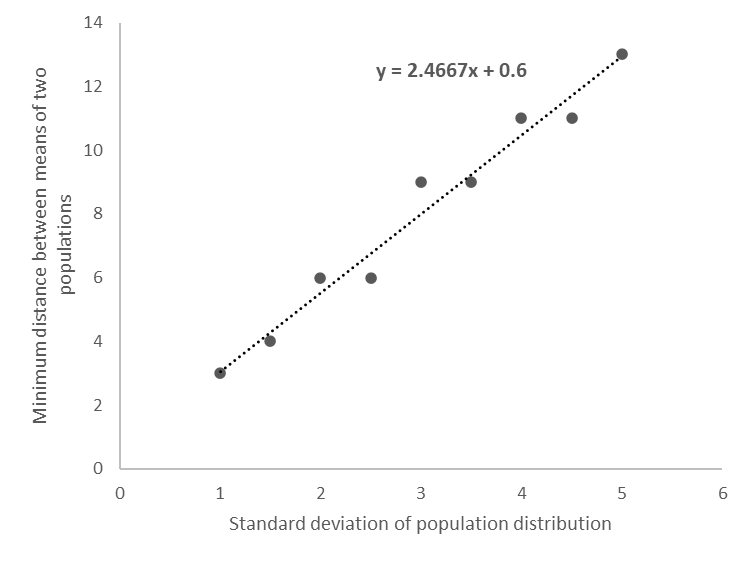


*Figure S5: Minimum distance between the means of the populations to be classified as two populations scales linearly with the standard deviations of the populations.*

Based on these results it can be inferred that two populations with mean values at least three standard deviations apart can be classified as two distinct populations. The minimum standard deviation seen in our experimental data for circular plasmids was 0.3 µm (Fig 3b) and for linear plasmids 2.2 µm (Fig 2i), which corresponds to minimum resolvable lengths between two sub-populations to be ~6 kbp and ~20 kbp respectively when two plasmids of similar lengths are present in the sample.


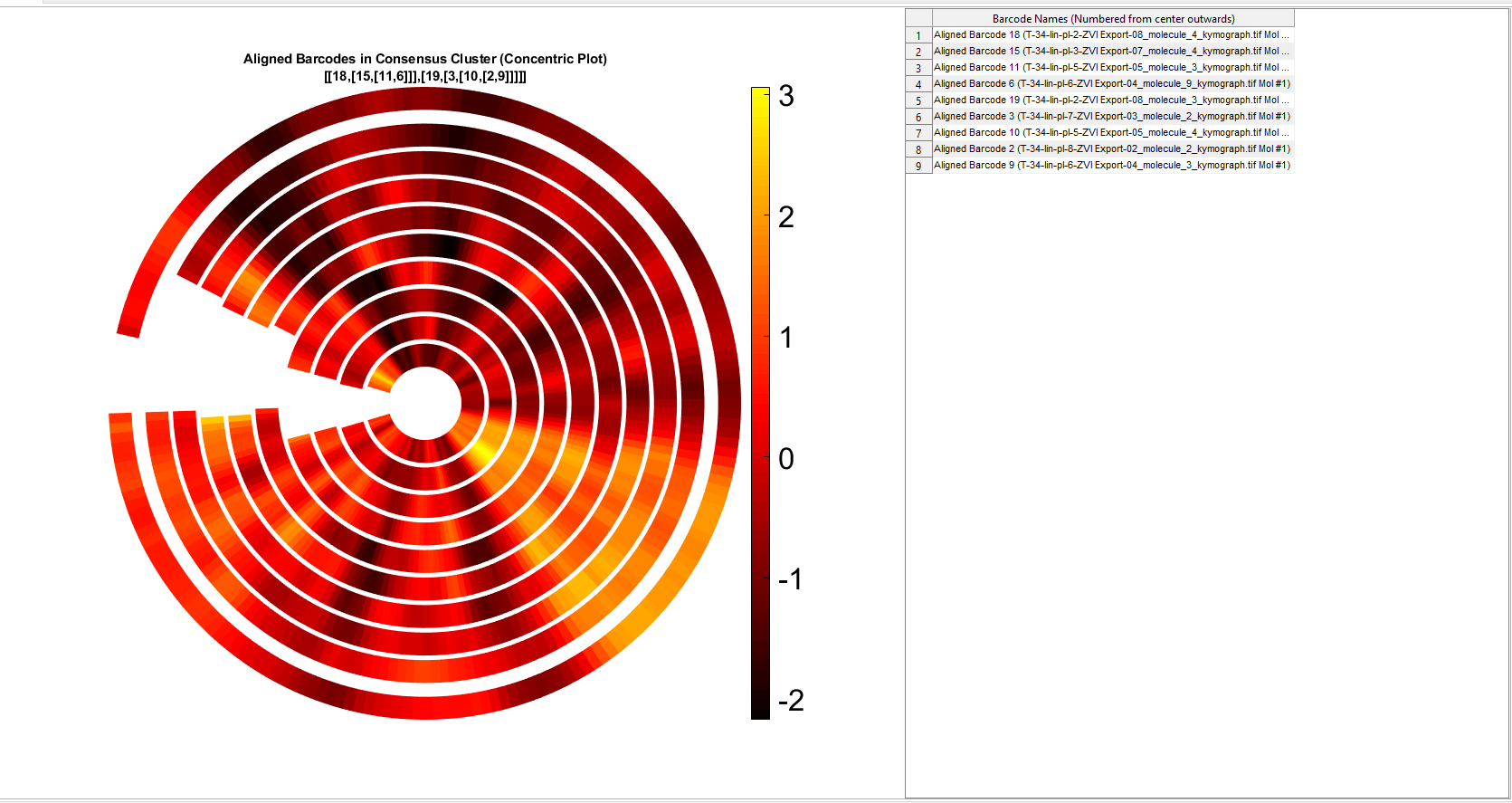


*Figure S6. Circular consensus of intensity profiles for nine plasmid molecules (each concentric circle represents one molecule) from the first plasmid preparation as shown in Fig. 2 in the main text. The plasmids were cut with Cas9 targeting the bla_NDM_ gene, stained with YOYO/netropsin and imaged in the nanochannels. All imaged molecules were aligned and molecules with cross-correlation greater than 0.7 were used to generate the plot. The discontinuity in the intensity profile is the result of Cas9 cut and the consensus observed in this data indicates presence of bla_NDM-1_ gene. Unpublished data.*


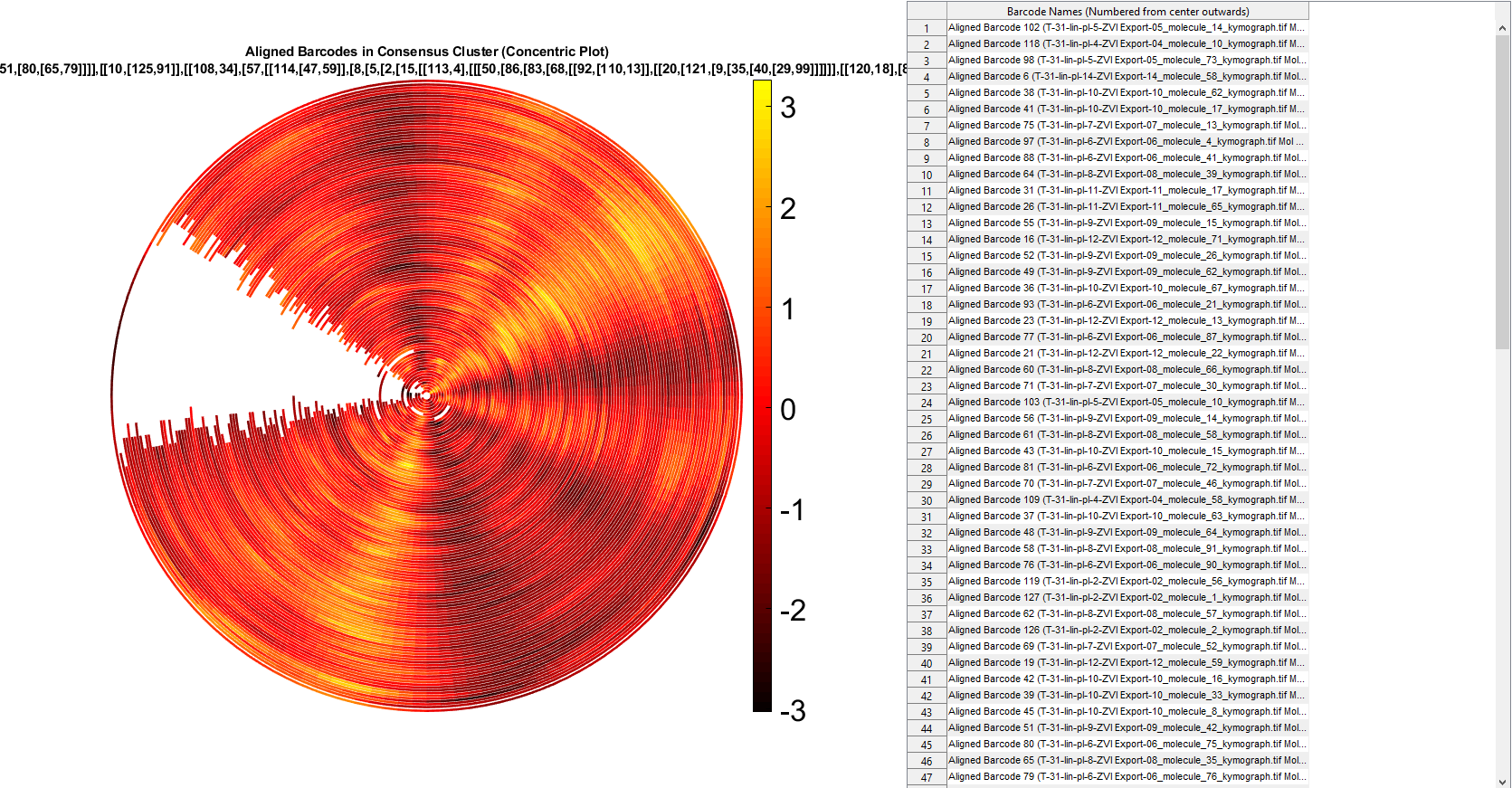


*Figure S7. Circular consensus of intensity profiles for plasmid molecules (each concentric circle represents one molecule) from the second plasmid preparation as shown in Fig. 2 in the main text. The plasmids were cut with Cas9 targeting the bla_NDM_ gene, stained with YOYO/netropsin and imaged in the nanochannels. All imaged molecules were aligned and molecules with cross-correlation greater than 0.7 were used to generate the plot. The discontinuity in the intensity profile is the result of Cas9 cut and the consensus observed in this data indicates presence of bla_NDM-1_ gene. This plasmid isolate is identified as P15F_2 in our published results on detection of AMR using optical mapping^6^.*


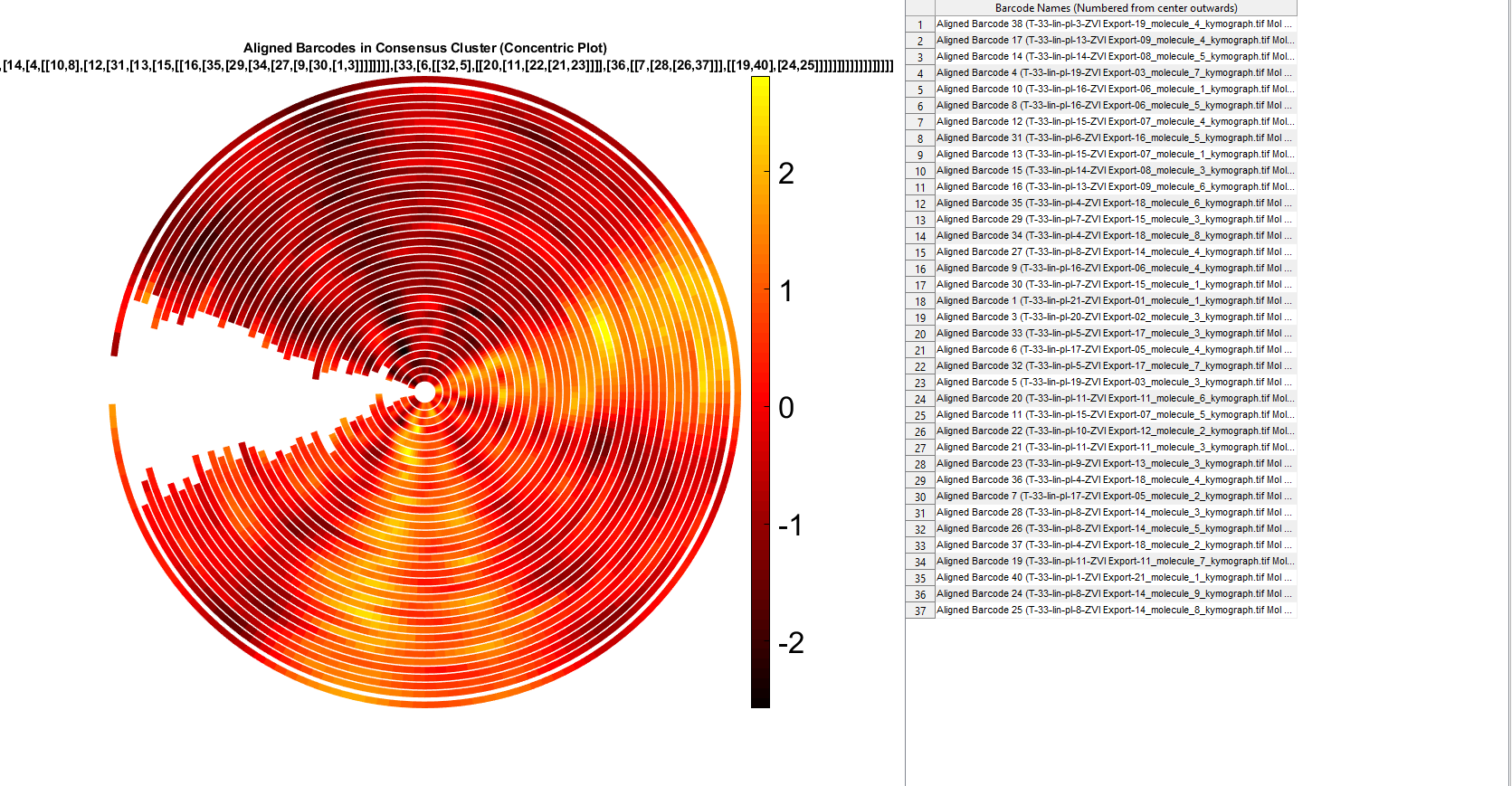


*Figure S8. The circular consensus of intensity profiles for thirty-seven plasmid molecules (each concentric circle represents one molecule) from the same plasmid preparation as shown in Fig. 3 in the main text. The plasmids were cut with Cas9 targeting the bla_NDM_ gene, stained with YOYO/netropsin and imaged in the nanochannels. All imaged molecules were aligned and molecules with cross-correlation greater than 0.7 were used to generate the plot. The discontinuity in the intensity profile is the result of the Cas9 cut and the consensus observed in this data indicates the presence of the bla_NDM-1_ gene. Unpublished data.*


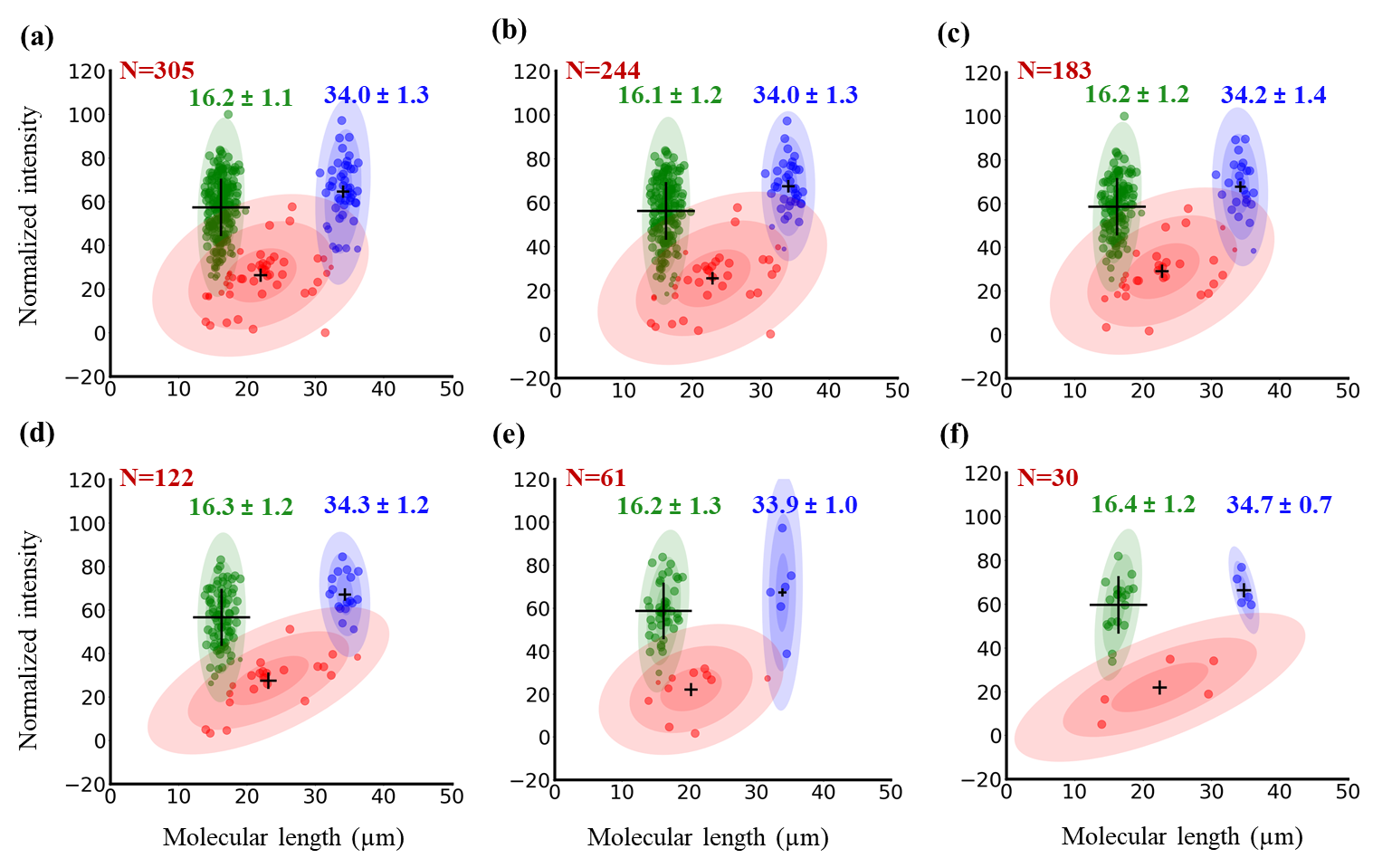


*Figure S9: Population distribution and clustering with decreasing number of data points. (a) Original data obtained from two plasmid population as shown in Fig 2d in the main text. (b)-(f) population clusters with randomly sampled data from (a). Number of data points, means and standard deviations for two plasmid populations are marked.*

| Table ST1: Fitting parameters for data presented in main Fig. 1 | | | | | |
| --- | --- | --- | --- | --- | --- |
|  |  | Mean Length | Std Dev Length | Relative weight | Sample N |
| Length histograms (Fig. 1g) | | | | | |
| Control | Black | 45.4 | 3.4 | 75.2% | 101 |
| Control | Black | 64.8 | 9.0 | 24.7% | 101 |
|  | | | | | |
| *bla*_NDM_ | Red | 46.7 | 5.1 | 52.0% | 286 |
| *bla*_NDM_ | Red | 98.3 | 10.1 | 32.4% | 286 |
| *bla*_NDM_ | Red | 68.2 | 8.5 | 15.6% | 286 |

| Table ST2: Fitting parameters for data presented in main Fig. 2 | | | | | | |
| --- | --- | --- | --- | --- | --- | --- |
|  |  | Mean Length | Std Dev Length | Relative weight | Sample N | |
| Length histograms (Fig.2 c) | | | | | | |
| Control | Green | 16.2 | 1.2 | 74.5% | 309 | |
| Control | Green | 23.0 | 2.1 | 7.6% | 309 | |
| Control | Green | 33.6 | 1.7 | 17.9% | 309 | |
|  | | | | | |  |
| *bla*_NDM_ | Purple | 16.3 | 1.3 | 11.1% | 290 | |
| *bla*_NDM_ | Purple | 28.1 | 5.6 | 32.6% | 290 | |
| *bla*_NDM_ | Purple | 32.7 | 2.3 | 56.2% | 290 | |
|  | | | | | |  |
|  | | | | | |  |
| Scatter plots and 2D clusters (Fig. 2 d-f) | | | | | |  |
| Control | Green | 16.2 | 1.2 | 69.9% | 309 | |
| Control | Red | 23.0 | 5.7 | 15.6% | 309 | |
| Control | Blue | 34.1 | 1.3 | 14.4% | 309 | |
|  | | | | | | |
| *bla*_NDM_ | Red | 21.6 | 5.8 | 27.3% | 290 | |
| *bla*_NDM_ | Blue | 30.4 | 4.0 | 15.9% | 290 | |
| *bla*_NDM_ | Purple | 32.7 | 2.5 | 56.7% | 290 | |
|  | | | | | |  |
| *bla*_CTX-M_ group 1 | Green | 17.3 | 1.9 | 40.1% | 129 | |
| *bla*_CTX-M_ group 1 | Purple | 33.7 | 4.7 | 47.9% | 129 | |
| *bla*_CTX-M_ group 1 | Blue | 34.4 | 4.0 | 11.9% | 129 | |
|  | | | | | | |
|  | | | | | | |
| Scatter plots and 2D clusters (Fig. 2 h-i) | | | | | | |
| Control | Green | 16.2 | 1.8 | 60.1% | 187 | |
| Control | Blue | 22.2 | 1.3 | 25.9% | 187 | |
| Control | Red | 33.5 | 2.9 | 14.1% | 187 | |
|  | | | | | | |
| *bla*_NDM_ | Blue | 18.7 | 3.5 | 47.5% | 272 | |
| *bla*_NDM_ | Red | 26.4 | 8.7 | 19.0% | 272 | |
| *bla*_NDM_ | Purple | 31.4 | 2.2 | 33.6% | 272 | |

| Table ST3: Fitting parameters for data presented in main Fig. 3 | | | | | |
| --- | --- | --- | --- | --- | --- |
|  |  | Mean Length | Std Dev Length | Relative weight | Sample N |
|  | | | | | |
| Control | Green | 12.3 | 0.3 | 26.9% | 170 |
| Control | Red | 13.7 | 2.8 | 30.6% | 170 |
| Control | Blue | 19.0 | 0.7 | 42.4% | 170 |
|  | | | | | |
| *bla*_NDM_ + *bla*_KPC_ | Green | 10.7 | 0.6 | 35.7% | 246 |
| *bla*_NDM_ + *bla*_KPC_ | Red | 17.5 | 4.2 | 28.7% | 246 |
| *bla*_NDM_ + *bla*_KPC_ | Purple | 34.0 | 2.7 | 35.6% | 246 |
|  | | | | | |
| *bla*_KPC_ | Green | 11.5 | 0.7 | 44.1% | 228 |
| *bla*_KPC_ | Red | 17.3 | 4.7 | 17.6% | 228 |
| *bla*_KPC_ | Blue | 17.6 | 1.1 | 38.2% | 228 |
|  | | | | | |
| *bla*_NDM_ | Green | 11.7 | 1.3 | 29.0% | 214 |
| *bla*_NDM_ | Red | 19.6 | 5.4 | 32.0% | 214 |
| *bla*_NDM_ | Purple | 37.2 | 2.4 | 38.9% | 214 |

| Table ST4: Fitting parameters for data presented in main Fig. 4 | | | | | |
| --- | --- | --- | --- | --- | --- |
|  |  | Mean Length | Std Dev Length | Relative weight | Sample N |
| Axiocam momo -202 Length histograms | | | | | |
| Control | Grey | 15.8 | 1.5 | 72.1% | 659 |
| Control | Grey | 21.2 | 3.0 | 16.9% | 659 |
| Control | Grey | 32.6 | 3.1 | 10.9% | 659 |
|  | | | | | |
| *bla*_NDM_ | Brown | 17.0 | 2.1 | 34.0% | 442 |
| *bla*_NDM_ | Brown | 24.7 | 3.0 | 25.9% | 442 |
| *bla*_NDM_ | Brown | 31.3 | 2.3 | 40.0% | 442 |
|  | | | | | |
| Axiocam momo -202 Scatter plots and 2D clusters | | | | | |
| Control | Green | 15.8 | 1.4 | 63.0% | 659 |
| Control | Red | 19.4 | 3.5 | 25.7% | 659 |
| Control | Blue | 32.4 | 3.5 | 11.3% | 659 |
|  | | | | | |
| *bla*_NDM_ | Green | 17.8 | 2.6 | 37.9% | 442 |
| *bla*_NDM_ | Blue | 26.3 | 5.4 | 17.4% | 442 |
| *bla*_NDM_ | Purple | 30.0 | 3.2 | 44.6% | 442 |
|  |  |  |  |  |  |
| Huawei P30 camera Length histograms | | | | | |
| Control | Grey | 16.7 | 1.6 | 68.5% | 955 |
| Control | Grey | 24.1 | 3.6 | 17.7% | 955 |
| Control | Grey | 35.9 | 3.5 | 13.7% | 955 |
|  | | | | | |
| *bla*_NDM_ | Brown | 16.5 | 1.7 | 29.8% | 465 |
| *bla*_NDM_ | Brown | 24.2 | 2.4 | 22.4% | 465 |
| *bla*_NDM_ | Brown | 31.8 | 2.7 | 47.7% | 465 |
|  | | | | | |
| Huawei P30 camera Scatter plots and 2D clusters | | | | | |
| Control | Green | 16.7 | 1.6 | 61.2% | 955 |
| Control | Red | 21.3 | 5.1 | 21.0% | 955 |
| Control | Blue | 33.3 | 5.8 | 17.8% | 955 |
|  | | | | | |
| *bla*_NDM_ | Green | 16.2 | 1.5 | 25.3% | 465 |
| *bla*_NDM_ | Blue | 25.4 | 5.0 | 34.3% | 465 |
| *bla*_NDM_ | Purple | 31.4 | 3.0 | 40.4% | 465 |

**References**

1. Müller, V.; Westerlund, F., Optical DNA mapping in nanofluidic devices: principles and applications. *Lab on a Chip* **2017,** *17* (4), 579-590.

2. Nyberg, L. K.; Quaderi, S.; Emilsson, G.; Karami, N.; Lagerstedt, E.; Müller, V.; Noble, C.; Hammarberg, S.; Nilsson, A. N.; Sjöberg, F., Rapid identification of intact bacterial resistance plasmids via optical mapping of single DNA molecules. *ScientificReports* **2016,** *6* (1), 1-10.

3. Müller, V.; Karami, N.; Nyberg, L. K.; Pichler, C.; Torche Pedreschi, P. C.; Quaderi, S.; Fritzsche, J.; Ambjörnsson, T.; Åhrén, C.; Westerlund, F., Rapid tracing of resistance plasmids in a nosocomial outbreak using optical DNA mapping. *ACS Infectious Diseases* **2016,** *2* (5), 322-328.

4. Müller, V.; Rajer, F.; Frykholm, K.; Nyberg, L. K.; Quaderi, S.; Fritzsche, J.; Kristiansson, E.; Ambjörnsson, T.; Sandegren, L.; Westerlund, F., Direct identification of antibiotic resistance genes on single plasmid molecules using CRISPR/Cas9 in combination with optical DNA mapping. *Scientific Reports* **2016,** *6* (1), 1-11.

5. Bikkarolla, S. K.; Nordberg, V.; Rajer, F.; Müller, V.; Kabir, M. H.; Sriram, K.; Dvirnas, A.; Ambjörnsson, T.; Giske, C. G.; Navér, L., Optical DNA mapping combined with Cas9-targeted resistance gene identification for rapid tracking of resistance plasmids in a neonatal intensive care unit outbreak. *MBio* **2019,** *10* (4).

6. KK, S.; Sewunet, T.; Wangchinda, W.; Tangkoskul, T.; Thamlikitkul, V.; Giske, C. G.; Westerlund, F., Optical DNA Mapping of Plasmids Reveals Clonal Spread of Carbapenem-Resistant Klebsiella pneumoniae in a Large Thai Hospital. *Antibiotics* **2021,** *10* (9), 1029.
